# Investigation of *Commiphora myrrha* (Nees) Engl. Oil and Its Main Components for Antiviral Activity

**DOI:** 10.3390/ph14030243

**Published:** 2021-03-09

**Authors:** Valentina Noemi Madia, Marta De Angelis, Daniela De Vita, Antonella Messore, Alessandro De Leo, Davide Ialongo, Valeria Tudino, Francesco Saccoliti, Giovanna De Chiara, Stefania Garzoli, Luigi Scipione, Anna Teresa Palamara, Roberto Di Santo, Lucia Nencioni, Roberta Costi

**Affiliations:** 1 Istituto Pasteur-Fondazione Cenci Bolognetti, Dipartimento di Chimica e Tecnologie del Farmaco, “Sapienza” Università di Roma, p.le Aldo Moro 5, I-00185 Rome, Italy; valentinanoemi.madia@gmail.com (V.N.M.); antonella.messore@uniroma1.it (A.M.); alessandro.deleo@uniroma1.it (A.D.L.); ialongo.1679357@studenti.uniroma1.it (D.I.); valeria.tudino@uniroma1.it (V.T.); luigi.scipione@uniroma1.it (L.S.); roberto.disanto@uniroma1.it (R.D.S.); roberta.costi@uniroma1.it (R.C.); 2 Laboratory Affiliated to Pasteur Italia-Fondazione Cenci Bolognetti, Department of Public Health and Infectious Diseases, “Sapienza” University of Rome, p.le Aldo Moro 5, I-00185 Rome, Italy; marta.deangelis@uniroma1.it (M.D.A.); annateresa.palamara@uniroma1.it (A.T.P.); lucia.nencioni@uniroma1.it (L.N.); 3 Department of Environmental Biology, “Sapienza” University of Rome, p.le Aldo Moro 5, I-00185 Rome, Italy; 4 D3 PharmaChemistry, Italian Institute of Technology, Via Morego 30, I-16163 Genova, Italy; francesco.saccoliti@iit.it; 5 Institute of Translational Pharmacology, National Research Council (CNR), Via del Fosso del Cavaliere 100, I-00133 Rome, Italy; giovanna.dechiara@ift.cnr.it; 6 Department of Drug Chemistry and Technologies, Sapienza University of Rome, p.le Aldo Moro 5, I-00185 Rome, Italy; stefania.garzoli@uniroma1.it

**Keywords:** *Commiphora myrrha*, sesquiterpenes, myrrh oil, vitamin E acetate, supercritical CO_2_ fluid extraction, HPLC, antiviral activity, influenza A H1N1 virus

## Abstract

The resinous exudate produced by *Commiphora myrrha* (Nees) Engl. is commonly known as true myrrh and has been used since antiquity for several medicinal applications. Hundreds of metabolites have been identified in the volatile component of myrrh so far, mainly sesquiterpenes. Although several efforts have been devoted to identifying these sesquiterpenes, the phytochemical analyses have been performed by gas-chromatography/mass spectrometry (GC–MS) where the high temperature employed can promote degradation of the components. In this work, we report the extraction of *C. myrrha* by supercritical CO_2_, an extraction method known for the mild extraction conditions that allow avoiding undesired chemical reactions during the process. In addition, the analyses of myrrh oil and of its metabolites were performed by HPLC and GC–MS. Moreover, we evaluated the antiviral activity against influenza A virus of the myrrh extracts, that was possible to appreciate after the addition of vitamin E acetate (α-tocopheryl acetate) to the extract. Further, the single main bioactive components of the oil of *C. myrrha* commercially available were tested. Interestingly, we found that both furanodienone and curzerene affect viral replication by acting on different steps of the virus life cycle.

## 1. Introduction

**Figure 1:**
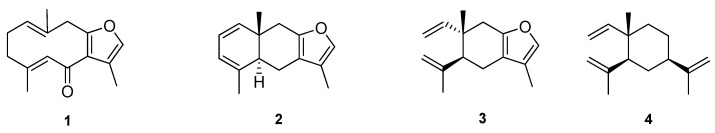
Structures of the main sesquiterpenes in myrrh oil: furanodienone (**1**), furanoeudesma-1,3-diene (**2**), curzerene (**3**) and β-elemene (**4**).

*Commiphora* genus (Burseraceae) includes more than 150 species especially occurring in northeastern Africa, southern Arabia and India [1]. The species *Commiphora myrrha* (Nees) Engl. produces a resinous exudate known as true myrrh that has been used for centuries as an embalming ointment, wound-healing remedy and for other medicinal applications [2]. Myrrh consists of alcohol-soluble resins and volatile oil together with a gum soluble in water containing polysaccharides, proteins and long chain aliphatic derivatives. The lipophilic part of myrrh is composed of steroids, sterols and terpenes [3]. To date, hundreds of metabolites have been identified in the volatile component of myrrh, where sesquiterpenes are the major components. Notably, furanosesquiterpenes such as furanoelemanes, furanoeudesmanes, and furanogermacranes are the characteristic constituents of myrrh oil [4]. As important components of volatile oil, sesquiterpenoids possess diverse biological activities, including antibacterial, antifungal, and antiparasitic properties. It is also worthy of note that recent papers reported the in vitro and in vivo analgesic and anticancer activities [5,6,7,8] of four main components of the volatile oil, namely furanodienone (**1**), furanoeudesma-1,3-diene (**2**), curzerene (**3**), and β-elemene (**4**) (Figure 1).

Some works [9,10] have also been devoted to the identification or characterization of these aforementioned sesquiterpenes but most of the phytochemical analyses have been performed by gas chromatography where the high temperature employed can promote degradation of the components, thus influencing the qualitative–quantitative composition of the mixture. For instance, the rearrangement of furanodiene to curzerene during the essential oil extraction process and/or during conventional gas chromatographic analysis, in which temperatures over 200 °C are employed, is reported [11].

Here we describe the extraction of *C. myrrha* by supercritical CO_2_, an extraction method that is well known for its many advantages in obtaining volatile extracts or aroma substances and, more generally, to extract lipophilic compounds from a wide variety of matrices. Indeed, in addition to being non-toxic and non-flammable, the mild extraction conditions allow avoiding undesired chemical reactions during the process. This leads to shorter extraction times without decomposing compounds vulnerable to high temperatures differently from hydro- or steam-distilled oils [12]. In the present work, the analysis of myrrh oil and the quantification of its metabolites were performed by HPLC, so as to avoid high temperatures also during the analytical process. Our interest in substances endowed with antiviral activity [13] and in the field of biologically active natural products [14] prompted us to evaluate the antiviral activity against influenza A virus of both the volatile myrrh extracts and of the single sesquiterpenes furanodienone (**1**), furanoeudesma-1,3-diene (**2**), curzerene (**3**) and β-elemene (**4**). In particular, we tested the myrrh extracts both alone and in combination with vitamin E acetate (α-tocopheryl acetate) in order to reduce the cytotoxic effects of the volatile oil. We decided also to investigate the activity of curzerene, furanodienone, furanoeudesma-1,3-diene and β-elemene **1**–**4**, being the main bioactive components of the volatile oil of *C. myrrha* [2] commercially available.

## 2. Results and Discussion

To determine the amount of the major sesquiterpenoids (**1**–**4**) in our extract, an HPLC analysis was carried out. The percentage of each sesquiterpene is reported in Table 1. It is possible to notice that furanoeudesma-1,3-diene is the most abundant while no furanodienone was detected in our extracts. Our results are in good agreement with those found by Marongiu et al. [12] who obtained supercritical fluid extraction (SCFE) myrrh oil made up of the following main components: furanoeudesma-1,3-diene (26.2%), curzerene (9.7%), and β-elemene (3.0%) without detecting any amount of furanodienone. A run at low temperature was used for the HPLC analysis to isolate compound **3** given that it could not be separated from other compounds in the mixture at higher temperatures.

To investigate the volatile composition of *C. myrrha* oil, gas-chromatography/mass spectrometry (GC/MS) technique was used. Twenty-two volatile compounds were identified representing an average of 99.9% of the oil composition and they are listed in Table 2. The most abundant component was furanoeudesma-1,3-dione (31.1%) followed by curzerene (23.1%), germacra-1(10),7,11-trien-15-oic acid, 8,12-epoxy-6-hydroxy-gamma-lactone (14.4%), lindestrene (11.9%) and other minor compounds.

**Figure 2:**
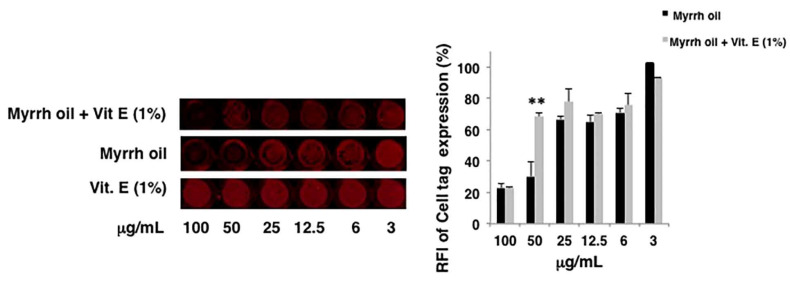
The mixture myrrh oil and vitamin E (Vit. E) acetate increased cell viability. A549 cells were treated with different concentrations (3–100 µg/mL) myrrh oil, Vit. E acetate (0.03–1 µg/mL) and a mixture of myrrh oil (3–100 µg/mL) with Vit. E acetate (0.03–1 µg/mL). Cells treated with DMSO were used as control. After 24 h, cell monolayers were fixed and stained with Cell Tag. The effect of compounds on the integrity of cell monolayers was analyzed by In-Cell Western assay (ICW), using LI-COR Image Studio Software. Images are representative of one experiment of two replicates, each performed in duplicate. The graph indicates the percentage of relative fluorescence intensity (RFI) of Cell Tag expressed on myrrh oil and myrrh oil + Vit. E acetate compared to Vit. E acetate is considered as 100%. Values are the mean ± S.D. of two replicates of one experiment performed in duplicate (*n* = 2); ** *p* < 0.01 vs. Vit. E acetate.

The extract obtained by supercritical fluid extraction (SCFE) using supercritical CO_2_ as solvent has been evaluated in vitro for its antiviral activity against influenza A Puerto Rico 8/34/H1N1 virus (PR8). First, we evaluated the eventual toxicity of compounds on the cell monolayers. Therefore, intact monolayers of A549 cells were treated with different concentrations (3–100 µg/mL) of myrrh oil. DMSO was used as control. The integrity of cell monolayers was evaluated by using a vital dye (Cell Tag) of cells and revealed by In-Cell Western assay (ICW). Myrrh oil alone was toxic at the highest concentrations: the integrity of cell monolayer was damaged of about 30, 71 and 76% compared to DMSO alone at 25, 50 and 100 µg/mL, respectively (data not shown). Due to the low stability of myrrh oil and its components in the air-oxygen environment, we hypothesized that the cytotoxicity of myrrh could be ascribed to the decomposition of the components of this oil. Thus, we decided to add a compound endowed with antioxidant properties to the extract. To this end, α-tocopheryl acetate and a mixture myrrh oil (3–100 µg/mL) plus α-tocopheryl acetate (1% *w*/*v*) were tested for their toxicity. We found that the addition of 1% vitamin E acetate to myrrh oil protected the cell monolayers starting from 50 µg/mL. Indeed, the percentage of relative fluorescence intensity (RFI) of Cell Tag indicated a recovery of cell integrity, reaching the same percentage of not toxic concentrations (Figure 2).

Therefore, since the addition of α-tocopheryl acetate (1% *w*/*v*) increased the viability of cells treated with the extract, in these conditions we could appreciate the antiviral activity of the mixture myrrh oil + α-tocopheryl acetate compared to DMSO treated–infected cells (control) measured by means of hemagglutination assay (HAU/mL). The mixture strongly reduced viral replication in a dose-dependent manner (see Table 3). The viral titer was determined by evaluating the presence of hemagglutinating activity in the supernatant of infected cells. When the cells were treated with myrrh oil alone or in combination with vitamin E (Vit. E), a decrease in viral titer expressed as HAU/mL values was measured, with no observed viral titer at concentration of 50 μg/mL. The Vit. E acetate alone did not interfere with viral replication: indeed, at concentrations ranging from 0.03 to 1 μg/mL, the values of viral titer were equal to 8 HAU/mL, as for the control (DMSO).

**Figure 3:**
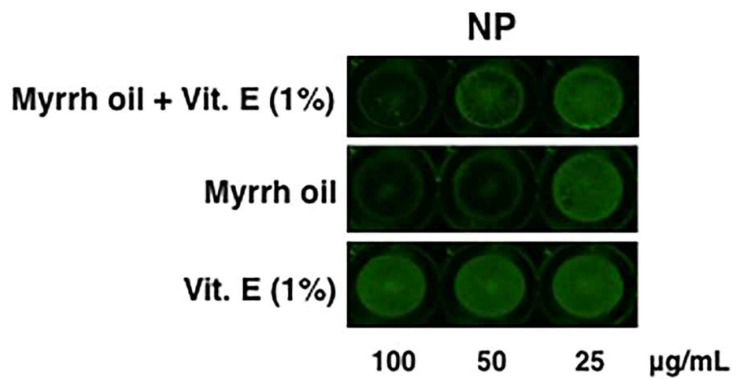
The mixture myrrh oil and Vit. E acetate inhibits influenza A virus replication. A549 cells were infected with PR8 and, after viral adsorption, treated with different concentrations myrrh oil (25, 50 and 100 µg/mL), Vit. E acetate (0.25–1 µg/mL) and myrrh oil (25, 50 and 100 µg/mL) in mixture with Vit. E acetate (0.25–1 µg/mL). Cells infected with PR8 and treated with DMSO were used as control. After 24 h, cell monolayers were fixed and the expression of viral nucleoprotein (NP) was analyzed by ICW, using LI-COR Image Studio Software. Images are representative of one experiment of two replicates, each performed in duplicate.

To confirm the antiviral activity of the mixture, cells were treated with the highest concentrations (25, 50 and 100 µg/mL) of myrrh oil alone, myrrh oil + Vit. E (1%) and Vit. E (1%). The expression of viral nucleoprotein (NP) was analyzed by ICW, a method that allows the evaluation of the expression of viral proteins directly on an infected monolayer [15]. As shown in Figure 3, the expression of NP was significantly reduced using myrrh oil at 25 and 50 µg/mL in mixture with Vit. E acetate, compared to Vit. E treated–infected cells (considered as 100%). In particular, the percentage of RFI was 89.7 ± 3% (* *p* < 0.05 vs. Vit E), 58.3 ± 5% (** *p* < 0.01 vs. Vit. E) and 23.4 ± 5% (** *p* < 0.01 vs. Vit. E) at 25, 50 and 100 µg/mL, respectively.

To further investigate the antiviral activity of myrrh oil components, we decided to test also furanodienone (**1**), furanoeudesma-1,3-diene (**2**), curzerene (**3**), and β-elemene (**4**) on A549 cells infected with PR8 virus. Indeed, these secondary metabolites are reported to be the main bioactive components of *C. myrrha* oil commercially available.

All the compounds were first tested for their potential toxicity on cell monolayer at different concentrations: furanodienone (**1**), curzerene (**3**) and β-elemene (**4**) at a range of 30–500 µg/mL; furanoeudesma-1,3-diene (**2**) at 2.5–30 µg/mL (due to the availability of a low amount of the standard compound). We found that compounds **1** and **3** showed toxicity at higher doses (starting from 100 µg/mL), while compound **4** was highly toxic at all the concentrations used. For this reason, the latter compound was excluded for the following experiments.

Compounds were added at different concentrations (30, 60 and 100 µg/mL) after the infection and maintained for the following 24 h. Viral replication was measured by means of HAU/mL. As shown in Table 4, compounds furanodienone (**1**) and curzerene (**3**) were effective at 60 and 100 µg/mL, even though the latter concentration was quite toxic for both compounds. In particular, for compound **1**, the viral titer was of 6 and 2 HAU/mL, at 60 and 100 µg/mL, respectively; for compound **3**, the viral titer was of 3 at 60 µg/mL, while no viral titer was observed at a concentration of 100 µg/mL. With regard to **2**, no inhibition was observed at any concentrations used (data not shown).

**Figure 4:**
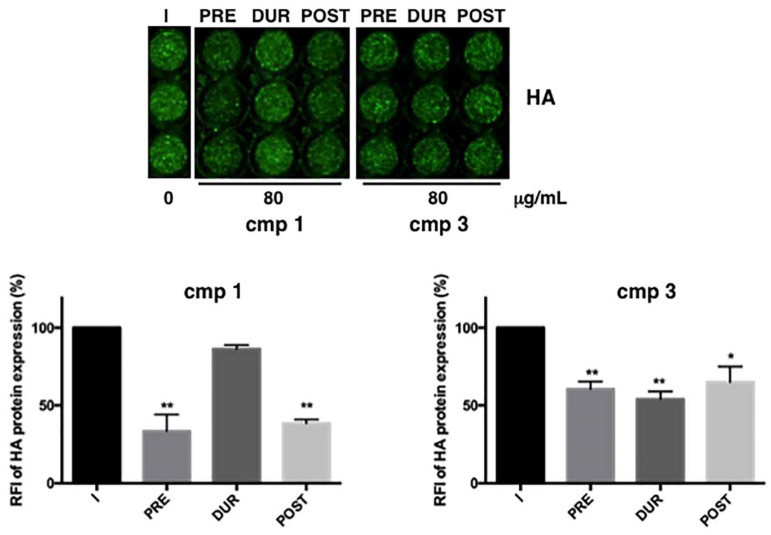
Compounds 1 and 3 affect different steps of virus lifecycle. A549 cells were infected with PR8 and treated or not with furanodienone (**1**) or curzerene (**3**) at different phases of the virus life cycle: the compounds were added for 2 h before (PRE); during viral adsorption for 1 h (DUR); immediately after viral adsorption and maintained for 24 h (POST). After 24 h infection, supernatants were recovered and used to infect a fresh monolayer of MDCK cells. The expression of viral Hemagglutinin (HA) was analyzed by ICW assay, using LI-COR Image Studio Software, as described in Methods. The percentage of RFI was calculated in comparison to Infected (I) cells (considered 100%). Values are the mean ± S.D. of three replicates (*n* = 3) of one out two experiments performed. Statistical significance of the data vs. I was defined as * *p* < 0.01 and ** *p* < 0.001.

Thus, the antiviral activity of the effective compounds **1** and **3** was analyzed by measuring the RFI of HA protein by ICW assay. However, the highest concentration of 100 µg/mL was quite toxic for both compounds, therefore, we chose a lower concentration to exclude that the antiviral activity may be in part due to the cytotoxicity of compounds. Therefore, to deepen the mechanism of action of furanodienone (**1**) and curzerene (**3**)**,** 80 µg/mL was added to cells at different steps of infection: only before infection (2 h, PRE), only during infection (1 h, DUR), immediately after infection and maintained for the following 24 h (POST). Compounds were also maintained for all the time of infection (before, during and after) but in this condition, they both became toxic for the cells, and data were not considered valid. As shown in Figure 4, both compounds were effective at specific phases: compound **1** was mainly effective when added PRE or POST (** *p* < 0.001); for compound **3,** although quite effective at all the phases, the most inhibition (46% compared to untreated cells) was reached during viral challenge (DUR). On the basis of these results, we hypothesize that compound **1** might act before infection by interfering with some cell factors on the plasma membrane or, due to the antioxidant activity of furanodienone [16], it may maintain reduced conditions into the cells making them more resistant to virus infection [17,18,19]. With regard to compound **3**, since it mainly acts during viral adsorption, it might interfere with the viral attachment or entry into the cells.

## 3. Materials and Methods

### 3.1. Materials

Oleo-gum-resin of *C. mhyrra* (origin Somalia) was purchased by Farmalabor (Italy). Before utilization, the material was ground with a Malavasi mill (Pinerolo, Italy) while care was taken to avoid overheating. The particles size was in the range 250–850 μm. CO_2_ (purity 99%) was supplied by SIO (Societa Italiana Ossigeno, Cagliari, Italy). Certified references furanoeudesma-1,3-diene and vitamin E acetate were purchased from Sigma-Aldrich (Milan, Italy), β-elemene, furanodienone and curzerene were purchased from Angene Chemicals (Nanjing, China). Acetonitrile HPLC grade was purchased from Sigma-Aldrich (Milan, Italy), and used without further purification. Water was purified before use by a Milli-Q plus 185 system (Milford, MA, USA).

### 3.2. Extraction

The powdered vegetable matter (1.30 kg) underwent the extraction process with an industrial-scale supercritical fluid extraction system SCFE-10 (Exenia Group, Pinerolo, TO, Italy). The supercritical CO_2_ extractions were performed as previously reported [20]. Briefly, in an apparatus equipped with 4200 mL extraction basket (with an internal diameter of 10 cm and an effective height of 42 cm) about 1.30 kg of myrrh was loaded at each run. Two filters of 80 μm were placed at both ends of the extraction basket and then placed in the 5 kg extractor vessel. The extraction parameters were set up as follows: temperature 65 °C, pressure 37 MPa and CO_2_ flow rate 18 kg CO_2_/h. The oil was collected in a glass container, previously autoclaved and weighted. An amount of 1.30 kg of powdered myrrh yielded 45.5 g of a brown oily extract at temperature 65 °C, pressure 37 MPa and CO_2_ flow rate 18 kg CO_2_/h.

### 3.3. Analysis

Method development for analysis of the extract was carried out with an HPLC system consisting of a Shimadzu LC-10AD pump, a SIL-10AD autosampler using a 200 μL sample loop, a CTO-10AC column oven, a SPD-10A detector. Data analyses were performed using the LC solutions software. The HPLC analyses were performed using an analytical column Waters Symmetry C18 (150 mm × 4.6 mm, 3.5 μm) by isocratic or in gradient elution. A concentrated stock solution of standards was prepared dissolving in acetonitrile an exactly weighted amount of the standards in a 5 mL volumetric flask; serial dilutions were performed obtaining solutions used to construct the calibration curves. A volume of 5 µL of each solution was injected. Peak area of each compounds was plotted vs. actual concentrations (mg/mL). Linearity was assessed through evaluation of the coefficient of determination (R^2^). Method details for each standard are reported below:

− Furanodienone (compound **1**): flow 0.5 mL/min, temperature 30 °C; gradient elution: 0–10 min (50% acetonitrile), 10–40 min (100% acetonitrile), 222 nm, tR = 14.10 min.
− Furanoeudesma-1,3-diene (compound **2**): flow 0.5 mL/min, temperature 30 °C; gradient elution: 0–15 min (80% acetonitrile), 15–30 min (100% acetonitrile), 222 nm, tR = 13.00 min.
− Curzerene (compound **3**): flow 0.5 mL/min, 18 °C, isocratic elution acetonitrile 70/water 30, 222 nm, tR = 30.02 min.
− β-elemene (compound **4**): flow 1 mL/min, 18 °C, isocratic elution acetonitrile 80/water 20, 205 nm, tR = 17.65 min.

An exactly weighted amount of myrrh extract was solubilized in acetonitrile (concentration ranging from 4 to 8 mg/mL) and 5 µL of this solution was injected for each HPLC analysis.

### 3.4. Analysis Gas-Chromatography–Mass Spectrometry (GC–MS) Analysis

The analysis of *C. myhrra* oil was carried out by a gas chromatograph equipped with flame ionization detector (FID) and a split–splitless injector and directly coupled to a mass spectrometer (MS) Perkin Elmer Clarus 500 model (Waltham, MA, USA). A Varian FactorFour VF-1 fused-silica capillary column (length 60 m × 0.32 mm ID × 1.0 μm film thickness) was used with helium as carrier gas (1.0 mL/min). A volume of 1 μL of EO oil was diluted in 1 mL of acetonitrile and the injection volume was 1μL. The chromatograph was equipped with a split/split less injector used in the split-less mode. The oven GC temperature program was as follows: from 70 to 250 °C at a rate of 5 °C/min, and held for 10 min; the injector temperature was 280 °C.

The electron-impact ionization mass spectrometer was operated as follows: ionization voltage, 70 eV; ion source temperature, 200 °C; scan mode, 40.0–450.0 mass range. GC–FID analyses were performed under the same operative conditions as described for the GC–MS measurements. The FID temperature was 280 °C. The identification of the components was assigned by matching their mass spectra with that reported on MS library search (Wiley and Nist). Furthermore, linear retention indices (LRIs) were calculated using a mixture of aliphatic hydrocarbons (*n*-alkanes C8-C30, Ultrasci) injected directly into GC injector at the same temperature program described above and compared with those reported in reference libraries. Two components were also identified by co-injection with an authentic sample. The semi-quantitative analysis was performed by normalizing the peak area generated in FID (%) without using corrections factors (RRFs). The analysis was repeated three times.

### 3.5. Cell Cultures and Viral Production

A549 (ATCC catalogue no. CCL-185), and MDCK (ATCC catalogue no. CCL-34) were used to determine the antiviral activity of myrrh oil and its metabolites. Cell lines were maintained in high glucose Dulbecco’s Modified Eagle’s Medium with sodium pyruvate and L-glutamine (DMEM; Euroclone, Milan, Italy), Minimum Essential Medium Eagle (EMEM; Euroclone, Milan, Italy) or Roswell Park Memorial Institute medium (RPMI-1640, Sigma, Milan, Italy) supplemented with 10% Fetal Bovine Serum (FBS; Euroclone, Milan, Italy) and 1% Penicillin/Streptomycin (Pen/Strep, Euroclone, Milan, Italy).

Allantoic cavities of 11-day-old embryonated chicken eggs were used to growth influenza virus A/Puerto Rico/8/34 H1N1 (PR8 virus). Viral suspension was inoculated in the allantoic cavity and incubated for 48 h at 37 °C, then infected eggs were maintained overnight at 4 °C. Subsequently, the allantoic fluid was collected and clarified by centrifugation (2500× *g* for 30 min). The recovered virus was used for the infection of MDCK and A549 cells. Allantoic fluid from uninfected eggs was used as reference for mock infection.

### 3.6. Cytotoxicity Assay

The cytotoxicity assay was evaluated on cell lines used for influenza virus infection by the MTT [3-(4,5-dimethylthiazol-2-yl)-2,5-diphenyltetrazolium bromide] assay. Briefly, cells were seeded in 96-well plates at a density of 2 × 10^4^ cells/well in 100 μL of complete RPMI medium without phenol red for 24 h at 37 °C. Subsequently, cell monolayers were treated or not with increasing concentrations (2.5–500 μg/mL) of compounds for 24 h at 37 °C. After 24 h, 10 μL of MTT solution (5 mg/mL) was added to each well for 4 h at 37 °C. Afterwards, each sample was acidified by adding 0.1 N HCl in isopropanol (100 μL/well) for 30 min in slow agitation to ensure that all formazan crystals were dissolved. Absorbance of samples was read at 570 nm, using an automatic plate reader (Multiskan EX, Ascent Software, Thermo Fisher Scientific, Milan, Italy). Untreated cells were used as control.

### 3.7. Hemagglutination (HAU) Assay

Virus production was evaluated in the supernatants of infected cells recovered 24 h after infection, by measuring the hemagglutinin units (HAU), using human type 0 Rh+ erythrocytes [19]. Stock solutions of compounds dissolved in DMSO were diluted in RPMI medium to final concentrations of 2.5–500 µg/mL. Compounds were added after the adsorption period and maintained in the culture media until the end of the experiments. The highest DMSO concentration present in the culture medium was 0.2%. Control cells were treated with DMSO alone at the same concentration present in the test substance being evaluated.

### 3.8. In Cell Western Assay

The ICW assay was performed using the Odyssey Imaging System (LI-COR, Lincoln, NE, USA) [19]. Briefly, A549 cells grown in 96-well plates (2 × 104 cells/well), either infected or mock-infected (Ctr) with PR8, were fixed with 4% formaldehyde, washed, permeabilized with 0.1% Triton X-100 and incubated with PBS containing Odyssey Blocking buffer (LI-COR Biosciences, Lincoln, NE, USA).

The cells were stained at 4 °C overnight with primary antibodies against influenza Nucleoprotein (NP) or Hemagglutinin (HA). After incubation, three washes with PBS plus 0.1% Tween 20 were performed and then the cells were stained with a mixture of fluorochrome-conjugated secondary antibodies (fluorescence emission at 800 nm), properly diluted in Odyssey blocking buffer and fluorochrome-conjugated Cell Tag (fluorescence emission at 700 nm), for 1 h at room temperature. Cell Tag was used as control of the integrity of cell monolayer. Subsequently, three washes with PBS plus 0.1% Tween 20 were performed and plates were analyzed by the Odyssey infrared imaging system (LI-COR). Integrated intensities of fluorescence were determined by the LI-COR Image Studio software and the relative fluorescence intensity (RFI) was expressed as percentage compared to untreated infected cells (100%).

### 3.9. Time-of-Addition Assay

A549 cells were seeded in 12-well plates at a density of 2.5 × 10^5^ cells/mL for 24 h at 37 °C, infected with PR8 and treated or not with furanodienone (**1**) or curzerene (**3**) (80 μg/mL) at different phases of the virus lifecycle, as indicated in the Results section. Untreated infected cells were used as control. Cell supernatants were recovered to infect fresh monolayer and to analyze viral protein expression by ICW assay. Statistical significance of the data was analyzed by means of a Student *t* test and *p* values < 0.05 were considered significant. Data were expressed as mean ± the standard deviation (SD).

## 4. Conclusions

In conclusion, we reported the extraction of *C. myrrha* by supercritical CO_2_, an extraction method that allowed avoiding undesired chemical reactions during the process. In addition, the analysis of the obtained myrrh oil and the quantification of its metabolites were performed by HPLC, so as to avoid high temperatures also during the analytical process. Interestingly, our study demonstrated that myrrh oil shows antiviral activity against influenza A Puerto Rico 8/34/H1N1 virus. Due the toxicity exerted by myrrh oil alone on the cell lines used for viral infection, we decided to add Vit. E acetate (1% *w*/*v*) that led to an increase in the viability of cells treated with the extract. It was also possible to appreciate the antiviral activity of myrrh oil plus Vit. E acetate. In particular, in the hemagglutination assay, the mixture of myrrh oil and the antioxidant compound showed a reduction in viral replication in a dose-dependent manner. Moreover, the expression of viral nucleoprotein was reduced using myrrh oil at 25 and 50 µg/mL in mixture with Vit. E acetate. Lastly, to investigate the antiviral activity of the main myrrh oil components, the single sesquiterpenes commercially available were tested. At non-toxic concentrations, the most active compounds, curzerene and furanodienone, proved to be effective at different steps of the virus lifecycle: furanodienone was mainly effective before and immediately after infection while curzerene was mainly active during viral adsorption. According to our results, myrrh and its metabolites may be useful to develop new antiviral agents active against influenza virus.

## Figures and Tables

**Table 1:** Percentage of compounds analyzed in the oil of *C. myrrha* extracted by supercritical fluid extraction (SCFE)-CO_2_ technique.

Compound	% (*w*/*w*)	LOQ	LOD
**1**	nd ^1^	3.62	1.19
**2**	10.48	1.61	0.53
**3**	2.35	3.81	1.26
**4**	1.04	2.59	0.86

^1^ Not detected; LOQ = limit of quantification (µg/mL): 10 × (SE/S); LOD = limit of detection (µg/mL): 3.3 × (SE/S); SE = standard deviation of regression of y intercept; S: slope of the linear calibration line.

**Table 2:** Chemical volatile composition (%) of myrrh oil.

N°	COMPONENT ^1^	LRI ^2^	LRI ^3^	MS ^4^	(%) ^5^
1	δ-elemene	1345	1347	+	0.8
2	copaene	1377	1379	+	0.4
3	β-elemene	1381	1387	+	3.9
4	γ-elemene	1443	1445	+	2.3
5	humulene	1470	1473	+	0.2
6	curzerene	1491	1495	+	23.1
7	germacrene D	1498	1500	+	0.3
8	δ-guajene	1501	1508	+	1.0
9	δ-cadinene	1505	1509	+	0.5
10	γ-cadinene	1533	1536	+	0.6
11	elemol	1539	1543	+	0.4
12	caryophyllene oxide	1585	1583	+	0.5
13	spathulenol	1603	1601	+	0.5
14	γ-eudesmol	1631	1630	+	0.3
15	isoserecenin	1790	1799 *	+	0.5
16	furanodienone	1870	§	+ ^a^	1.7
17	furanoeudesma-1,3-dione	2155	§	+ ^a^	31.1
18	lindestrene	2162	§	+	11.9
19	germacra-1(10),7,11-trien-15-oic acid, 8,12-epoxy-6-hydroxy-gamma-lactone	2170	§	+	14.4
20	1-(2,4-diemthylphenyl)-2-(2-furyl) cyclopropane	2180	§	+	2.6
21	cycloisolongifolene, 8,9-dehydro-9-formyl-	2200	§	+	2.4
22	8-hydroxy-3,8a-dimethyl-5-methylene-2-oxododecahydronaphto[2,3-b]furan-4-yl acetate	2210	§	+	0.5
	TOTAL (%)				99.9

^1^ The components are reported according their elution order on an apolar column; ^2^ linear retention indices measured on an apolar column; ^3^ linear retention indices from the literature; §: linear retention indices (LRI) not available for apolar column; *: Kovats Index RI; ^4^ MS: identification by comparison of the mass spectra with those present in the libraries; ^a^: identification confirmed by co-injection of an authentic sample; ^5^ percentage mean values of *C. myrrha* oil components (%).

**Table 3:** Antiviral activity of *C. myrrha* SCFE-CO_2_ extract alone and in combination with Vit. E acetate.

	Viral Titer (HAU/mL) ^1^						
Myrrh oil	8	4	4	4	1.5 ^2^	0	0
Myrrh oil + Vit. E acetate (1% *w*/*v*)	8	4	4	4	4	0	0
DMSO	8	8	8	8	8	8	8
Myrrh oil concentration (μg/mL)	0	3	6	12.5	25	50	100

^1^ Viral titer was measured by hemagglutination assay (HAU/mL) in the supernatant of infected cells treated or not with the compounds alone or in combination with Vit. E (1%), DMSO was used as control. Viral titer for Vit. E acetate was equal to 8 HAU/mL at all the tested concentrations (0.03–1 μg/mL); ^2^ SD ± 0.5.

**Table 4:** Antiviral activity of **1** and **3.**

Compound	Viral Titer (HAU/mL) ^1^			
**1**	8	8	6 ^2^	2
**3**	8	8	3 ^3^	0
Concentration (μg/mL)	0	30	60	100

^1^ Viral titer was measured by hemagglutination assay (HAU/mL) in the supernatant of infected cells treated or not with the compounds; ^2^ SD ± 2; ^3^ SD ± 01.

## Data Availability

The data presented in this study are available on request from the corresponding author.
